# Supplementary figures and images for: Network Topologies and Dynamics Leading to Endotoxin Tolerance and Priming in Innate Immune Cells
Source: PLoS Comput Biol. 2012 May 17;8(5):e1002526. doi: 10.1371/journal.pcbi.1002526 (PMC3355072; doi:10.1371/journal.pcbi.1002526)

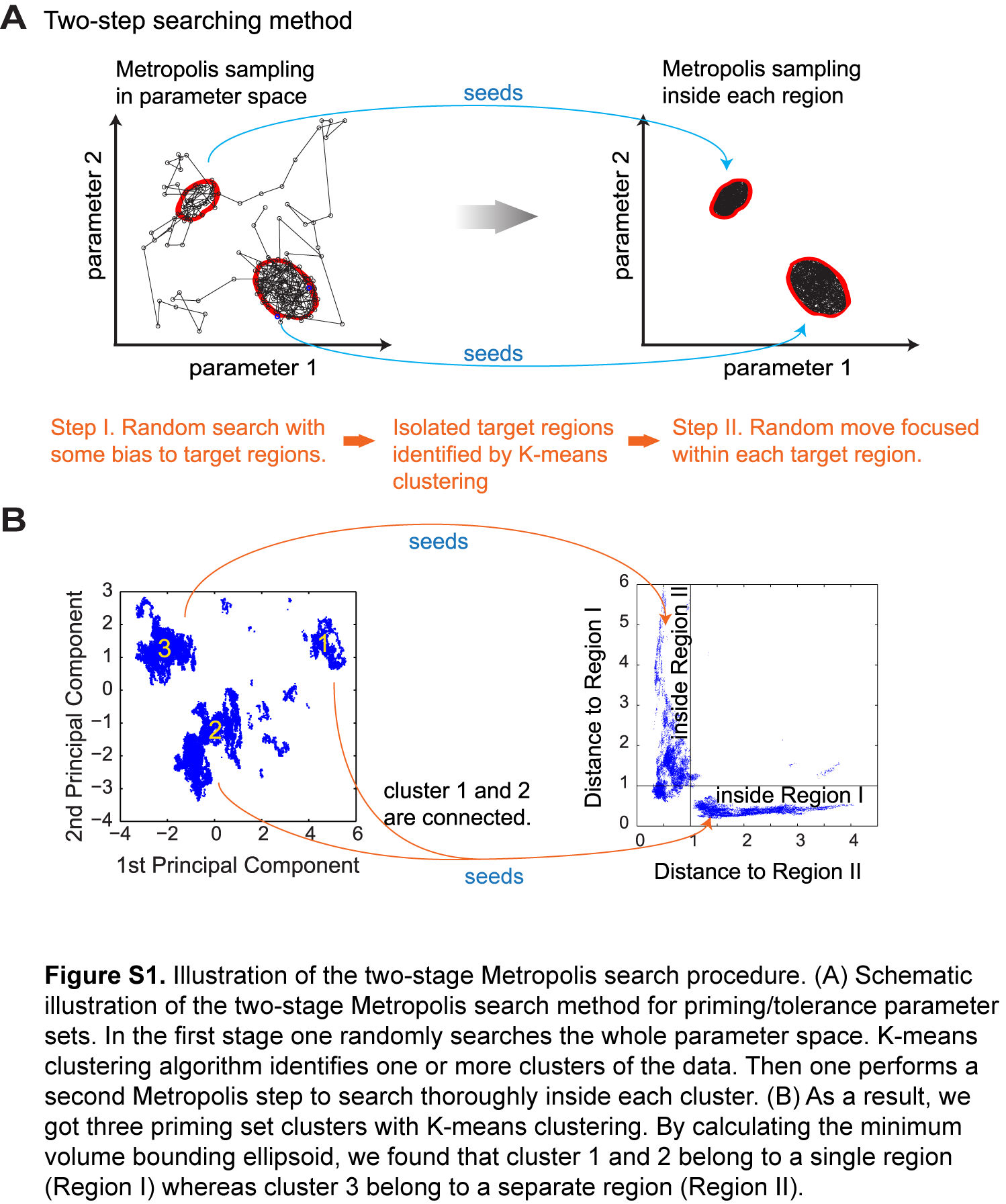

Supplement: Figure S1 — Illustration of the two-stage Metropolis search procedure. (A) Schematic illustration of the two-stage Metropolis search method for priming/tolerance parameter sets. In the first stage one randomly searches the whole parameter space. K-means clustering algorithm identifies one or more clusters of the data. Then one performs a second Metropolis step to search thoroughly inside each cluster. (B) As a result, we got three priming set clusters with K-means clustering. By calculating the minimum volume bounding ellipsoid, we found that cluster 1 and 2 belong to a single region (Region I) whereas cluster 3 belong to a separate region (Region II). (TIF) [file pcbi.1002526.s001.tif]
