# Supplementary material for: Network Topologies and Dynamics Leading to Endotoxin Tolerance and Priming in Innate Immune Cells
Source: PLoS Comput Biol. 2012 May 17;8(5):e1002526. doi: 10.1371/journal.pcbi.1002526 (PMC3355072; doi:10.1371/journal.pcbi.1002526)
Supplement: Figure S2 — Distribution of change in x 2's initial condition prior to HD without or without priming treatment. Both PS and AI show considerable increase in x 2 in the primed system. PDF: probability distribution function. (PDF) [file pcbi.1002526.s002.pdf]

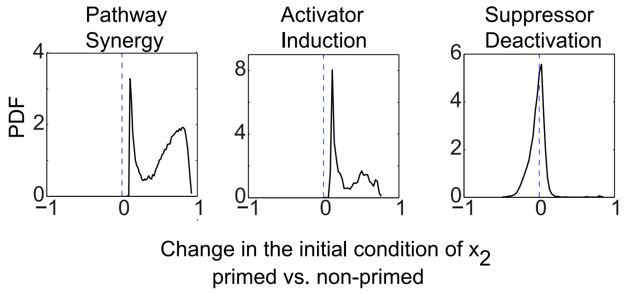

Figure S2. Distribution of change in x2's initial condition prior to HD without or without priming treatment. Both PS and AI show considerable increase in x2 in the primed system. PDF: probability distribution function.
