# Supplementary material for: Network Topologies and Dynamics Leading to Endotoxin Tolerance and Priming in Innate Immune Cells
Source: PLoS Comput Biol. 2012 May 17;8(5):e1002526. doi: 10.1371/journal.pcbi.1002526 (PMC3355072; doi:10.1371/journal.pcbi.1002526)
Supplement: Figure S3 — Statistical method used to identify backbone motifs from priming/tolerance data. (PDF) [file pcbi.1002526.s003.pdf]

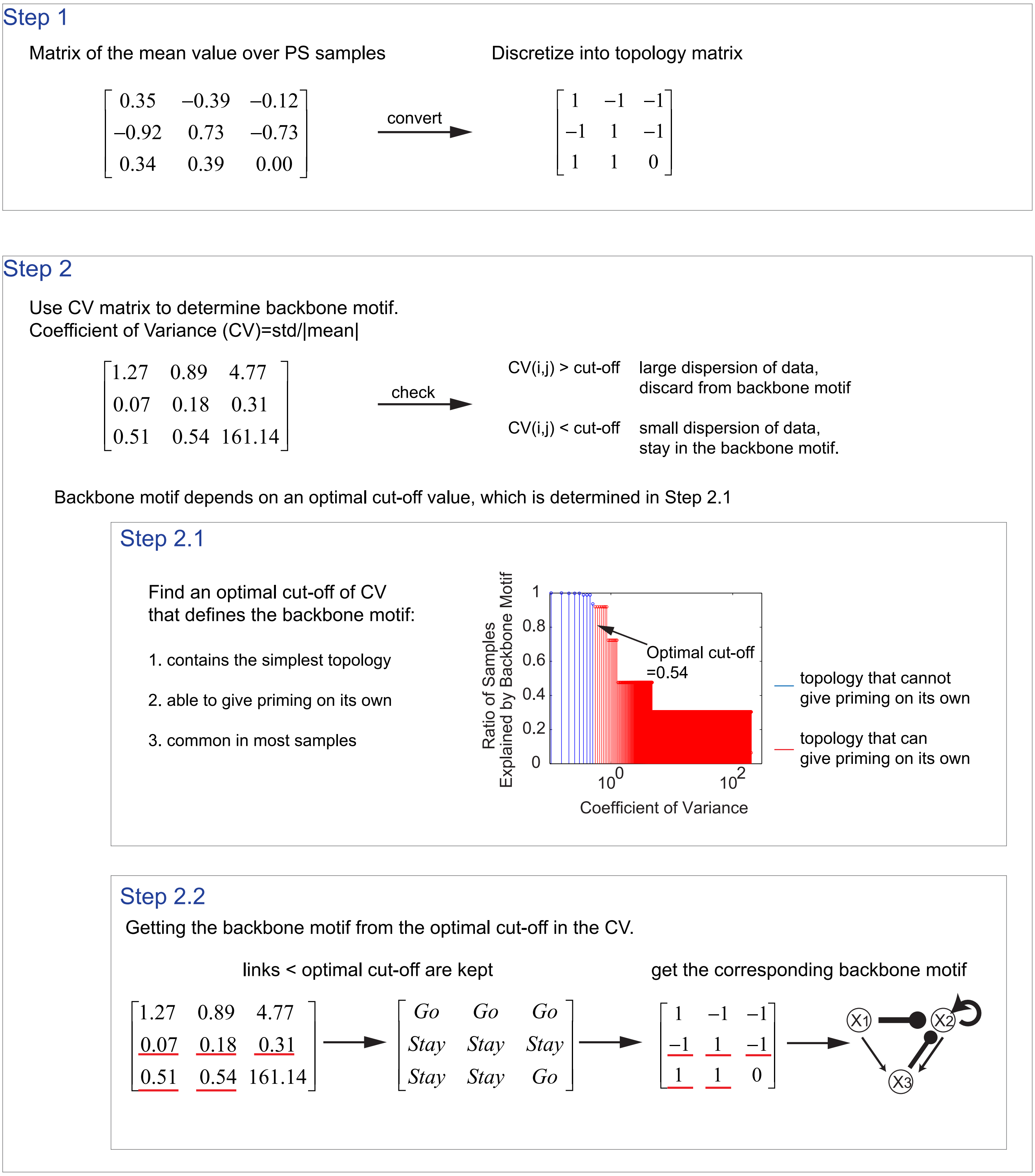

Figure S3. Statistical method used to identify backbone motifs from priming/tolerance data.
