# Supplementary material for: Network Topologies and Dynamics Leading to Endotoxin Tolerance and Priming in Innate Immune Cells
Source: PLoS Comput Biol. 2012 May 17;8(5):e1002526. doi: 10.1371/journal.pcbi.1002526 (PMC3355072; doi:10.1371/journal.pcbi.1002526)
Supplement: Figure S4 — Parameter correlations highlight the backbone motifs of each priming mechanism: (A) Pathway Synergy, (B) Suppressor Deactivation, and (C) Activator Induction. (PDF) [file pcbi.1002526.s004.pdf]

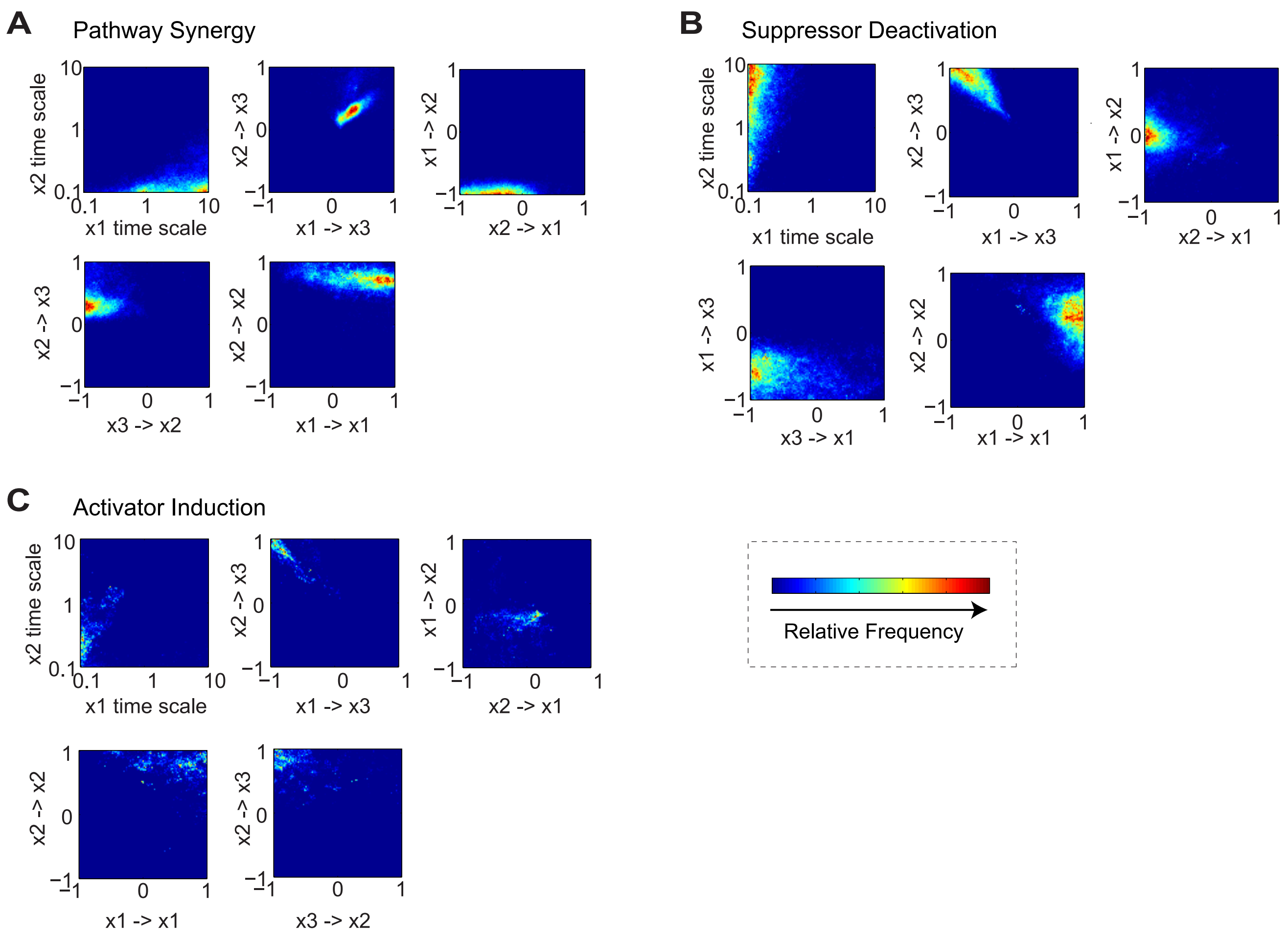

Figure S4. Parameter correlations highlight the backbone motifs of each priming mechanism. (A) Pathway Synergy, (B) Suppressor Deactivation, and (C) Activator Induction.
