# Supplementary material for: Network Topologies and Dynamics Leading to Endotoxin Tolerance and Priming in Innate Immune Cells
Source: PLoS Comput Biol. 2012 May 17;8(5):e1002526. doi: 10.1371/journal.pcbi.1002526 (PMC3355072; doi:10.1371/journal.pcbi.1002526)
Supplement: Figure S5 — Typical time course and corresponding trajectory in the phase space. (A) bistable case of AI mechanism. (B) bistable case of SD mechanism. Refer to Figure 3 of the main text for the time course trajectories in other cases. (PDF) [file pcbi.1002526.s005.pdf]

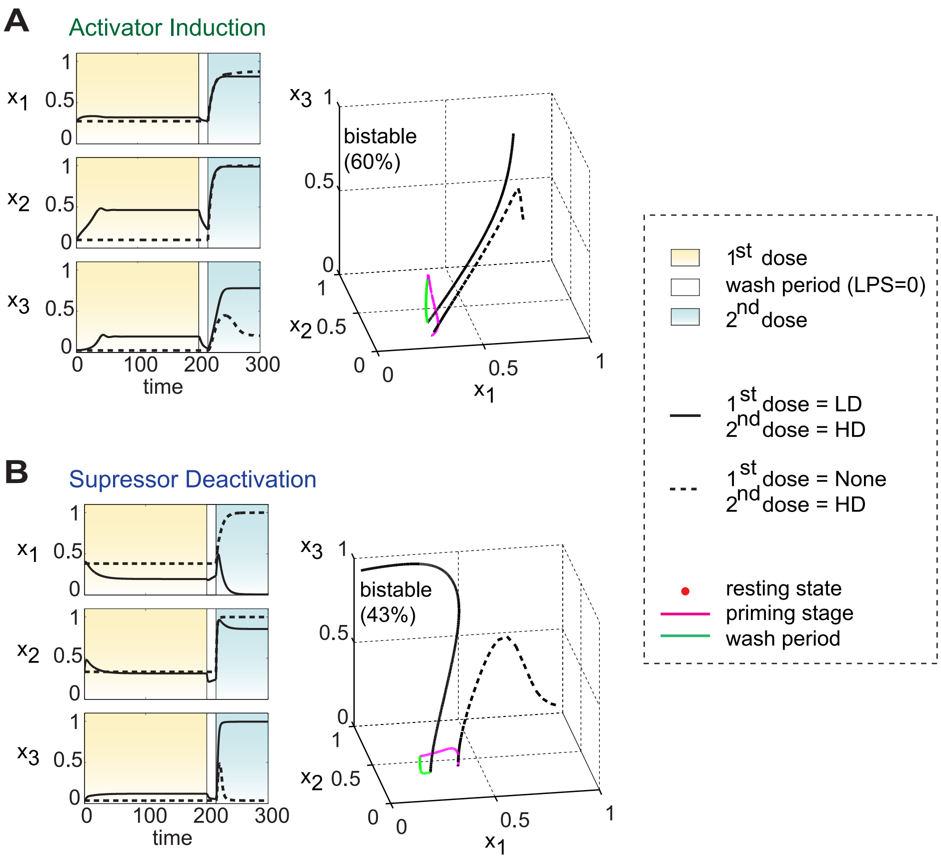

Figure S5. Typical time course and corresponding trajectory in the phase space. (A) bistable case of AI mechanism. (B) bistable case of SD mechanism. Refer to Figure 3 of the main text for the time course trajectories in other cases.
