# Supplementary material for: Network Topologies and Dynamics Leading to Endotoxin Tolerance and Priming in Innate Immune Cells
Source: PLoS Comput Biol. 2012 May 17;8(5):e1002526. doi: 10.1371/journal.pcbi.1002526 (PMC3355072; doi:10.1371/journal.pcbi.1002526)
Supplement: Figure S6 — Change in the robustness rank as a result of variations in the topology cut-off. SD datasets are used as an example. The robustness rank is calculated based on density (top panel) or sample frequency (lower panel) of the unique topologies. Changes in the robustness rank is compared with 10% (left column), 30% (center column), and 50% (right column) variation in the topology cut-off τ0 = 0.1. (PDF) [file pcbi.1002526.s006.pdf]

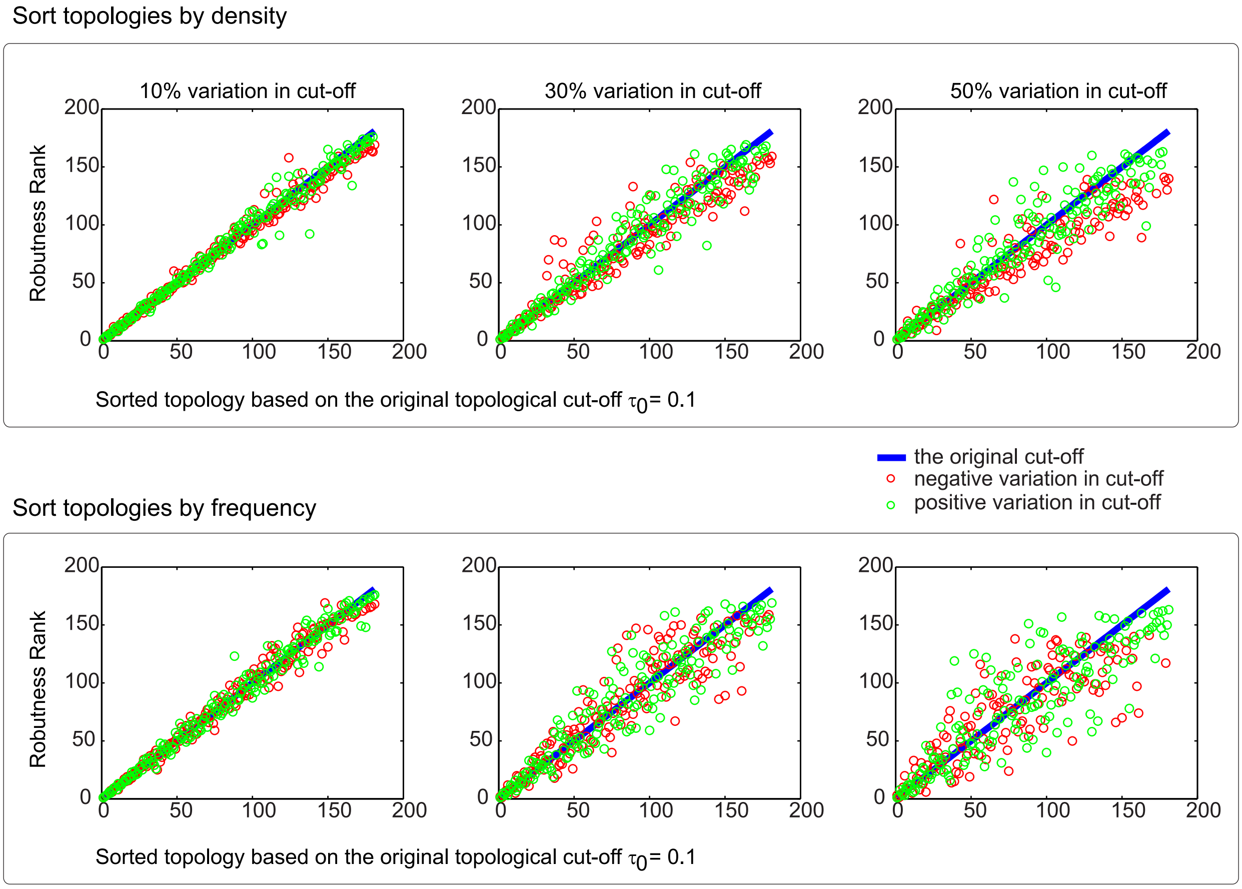

Figure S6. Change in the robustness rank as a result of variations in the topology cut-off. SD datasets are used as an example. The robustness rank is calculated based on density (top panel) or sample frequency (lower panel) of the unique topologies. Changes in the robustness rank is compared with 10% (left column), 30% (center column), and 50% (right column) variation in the topology cut-off $\tau 0$=0.1.
