# Supplementary material for: Network Topologies and Dynamics Leading to Endotoxin Tolerance and Priming in Innate Immune Cells
Source: PLoS Comput Biol. 2012 May 17;8(5):e1002526. doi: 10.1371/journal.pcbi.1002526 (PMC3355072; doi:10.1371/journal.pcbi.1002526)
Supplement: Figure S7 — Topologies of the PS and AI mechanisms. (A) The topology density distribution for the PS mechanism. (B) Top six PS topologies and the backbone motif. (C) The topology density distribution for the AI mechanism. (D) Top six AI topologies and the backbone motif. Line widths are proportional to the mean value of samples of the corresponding topology. Dashed lines denote the additional links present in the top topologies but absent in the backbone motif. (PDF) [file pcbi.1002526.s007.pdf]

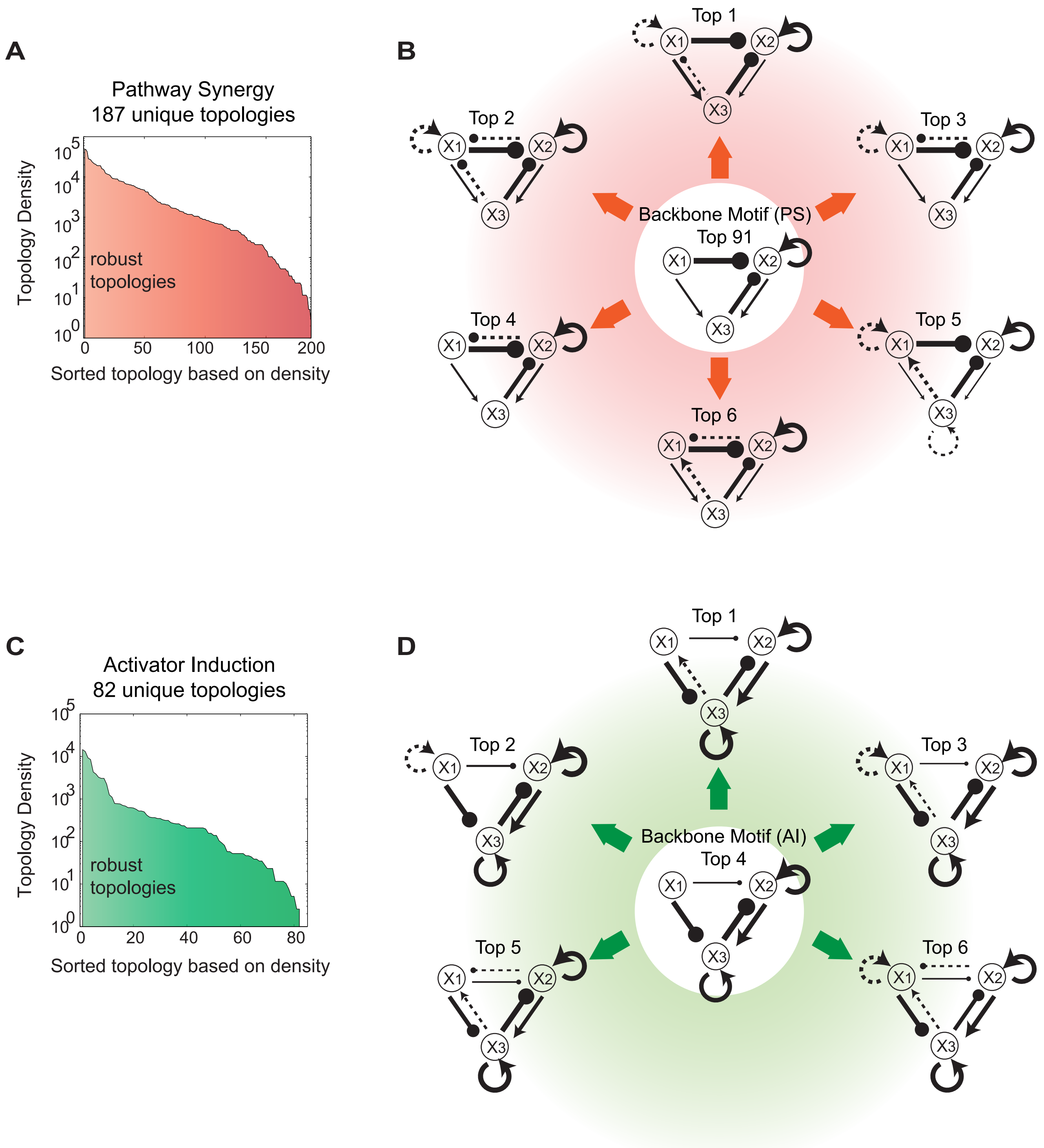


**Figure S7**. Topologies of the PS and AI mechanisms. (A) The topology density distribution for the PS mechanism. (B) Top six PS topologies and the backbone motif. (C) The topology density distribution for the AI mechanism. (D) Top six AI topologies and the backbone motif. Line widths are proportional to the mean value of samples of the corresponding topology. Dashed lines denote the additional links present in the top topologies but absent in the backbone motif.
