# Supplementary material for: Network Topologies and Dynamics Leading to Endotoxin Tolerance and Priming in Innate Immune Cells
Source: PLoS Comput Biol. 2012 May 17;8(5):e1002526. doi: 10.1371/journal.pcbi.1002526 (PMC3355072; doi:10.1371/journal.pcbi.1002526)
Supplement: Figure S8 — Parameter correlation and compensation affects the robustness of the model. A) Correlation matrix calculated based on the samples of each priming mechanism. The p-value is smaller than 0.05 except where marked. B) The parameter compensation mechanism is illustrated by the 2D correlation histogram of the SD samples (left) and the corresponding connection diagrams (right). (PDF) [file pcbi.1002526.s008.pdf]

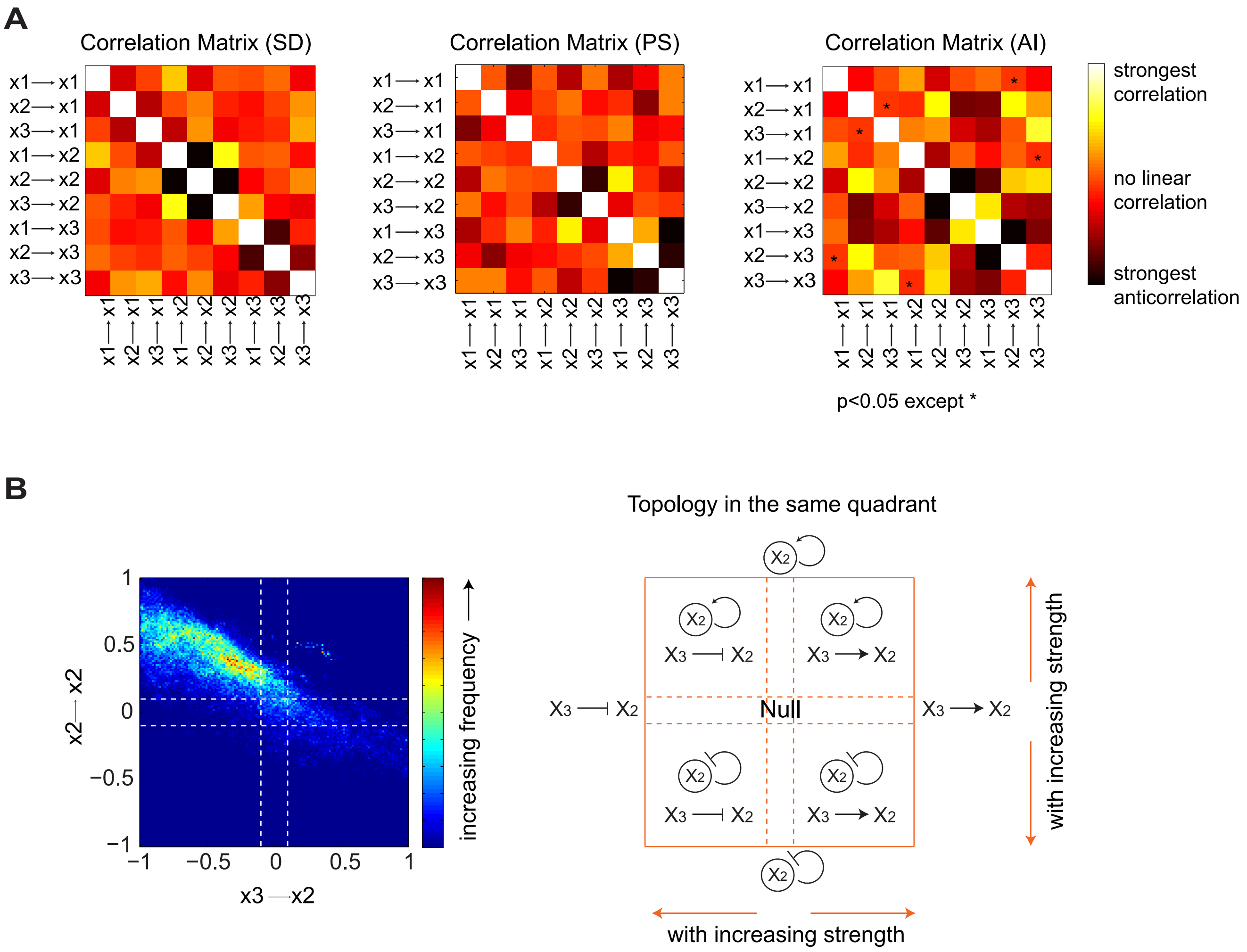

Figure S8. Parameter correlation and compensation affects the robustness of the model. (A) Correlation matrix calculated based on the samples of each priming mechanism. The p-value is smaller than 0.05 except where marked. (B) The parameter compensation mechanism is illustrated by the 2D correlation histogram of the SD samples (left) and the corresponding connection diagrams (right).
