# Supplementary material for: Network Topologies and Dynamics Leading to Endotoxin Tolerance and Priming in Innate Immune Cells
Source: PLoS Comput Biol. 2012 May 17;8(5):e1002526. doi: 10.1371/journal.pcbi.1002526 (PMC3355072; doi:10.1371/journal.pcbi.1002526)
Supplement: Table S1 — Criteria identifying priming and tolerance for a given parameter set. (PDF) [file pcbi.1002526.s009.pdf]

Table S1. Criteria identifying priming and tolerance for a given parameter set *x*.

A Good set of	Single LD	Single HD	LD+HD	HD+HD
**Priming**	$R_{LD}(x) < \delta_{LD}$	$R_{HD}(x) \geq \delta_{HD}$	$R_{LD+HD}(x)/R_{HD}(x) \geq \lambda$	-
**Tolerance**	$R_{LD}(x) < \delta_{LD}$	$R_{HD}(x) \geq \delta_{HD}$	-	$R_{HD}(x)/R_{HD+HD}(x) \geq \lambda$
Description	LD signal stimulates small response.	HD signal stimulates large response.	Two sequential signals (LD followed by HD) gives a larger response than a single HD.	Two sequential signals (HD followed by HD) gives a smaller response than a single HD.

R denotes the maximum response of "cytokine" x_3 under a specific stimulation protocol. LD: low dose; HD: high dose; LD+HD: LD followed by HD with maximum response measured in the HD period; HD+HD: HD followed by HD with maximum response measured in the second HD period. δ_{LD} and δ_{HD} denote the threshold of response under LD and HD, respectively. $\lambda > 1$ is the threshold of fold-change in the maximum response. The values we have chosen for these parameters (LD=0.1, HD=1, $\delta_{LD}=\delta_{HD}=0.3$, $\lambda=1.5$) are in qualitative agreement with experimental observations.
