# Supplementary material for: Network Topologies and Dynamics Leading to Endotoxin Tolerance and Priming in Innate Immune Cells
Source: PLoS Comput Biol. 2012 May 17;8(5):e1002526. doi: 10.1371/journal.pcbi.1002526 (PMC3355072; doi:10.1371/journal.pcbi.1002526)
Supplement: Table S2 — Parameter sets used to generate time course and phase-space trajectory in Figure 3 and Figure S5. (PDF) [file pcbi.1002526.s010.pdf]

Table S2. Parameter sets used to generate time course and phase-space trajectory in Figure 3 and Figure S5.

	**PS**	**PS**	**AI**	**AI**	**PS**	**PS**
	bistable	monostable	bistable	monostable	bistable	monostable
ω_{11}	0.26	0.19	-0.54	0	0.86	0.84
ω_{12}	-0.92	-0.27	0.05	-0.11	-0.78	-0.90
ω_{13}	0.61	0.23	-0.24	0.04	-0.86	-0.36
ω_{21}	-0.95	-0.93	-0.61	-0.52	0.36	0.08
ω_{22}	0.53	0.54	0.99	0.95	0.06	0.16
ω_{23}	-0.54	-0.35	-0.69	-0.89	-0.53	-0.45
ω_{31}	0.18	0.18	-0.80	-0.75	-0.96	-0.85
ω_{32}	0.47	0.27	0.83	0.82	0.89	0.93
ω_{33}	0.12	0.40	0.69	0.77	0.61	0.54
γ_1	1.56	0.43	0.10	0.11	0.14	0.15
γ_2	0.11	0.11	0.19	0.16	0.76	9.96
γ_3	1.00	1.00	1.00	1.00	1.00	1.00
σ_1	6.84	8.00	4.36	4.37	7.96	5.36
σ_2	7.19	8.00	6.55	6.89	6.33	5.50
σ_3	6.00	8.00	6.00	6.00	6.00	6.00
ω_{10}	-0.75	-0.50	-0.07	-0.22	-0.10	-0.15
ω_{20}	-0.25	-0.25	-0.25	-0.25	-0.25	-0.25
ω_{30}	-0.50	-0.50	-0.50	-0.50	-0.50	-0.50
