# Supplementary material for: Network Topologies and Dynamics Leading to Endotoxin Tolerance and Priming in Innate Immune Cells
Source: PLoS Comput Biol. 2012 May 17;8(5):e1002526. doi: 10.1371/journal.pcbi.1002526 (PMC3355072; doi:10.1371/journal.pcbi.1002526)
Supplement: Table S3 — Experimental literatures supporting the network details in Figure 8. (PDF) [file pcbi.1002526.s011.pdf]

Table S3. Experimental literature supporting the network details in Figure 8.

**Figure 8 Panel**	**Source**	**Target**	**Regulatory Type**	**Reference**	**Comment**
A	TLR4	IRAK	Activation	[1,2]	
A	IRAK	P38	Activation	[3,4]	
A	P38	IL-10	Transcription	[5]	
A	IL-10	IL-12	inhibition	[6,7]	
A	IL-10	TNFα	inhibition	[8,9]	
A	IRAK	AP-1	Activation	[10]	
A	AP-1	IL-12	Transcription	[11,12]	
A	IL-12	TNFα	Transcription	[9,13]	
A	TNFα	TNFα	Positive auto-regulation involving an autocrine loop	[14]	
A	IL-12	IL-12	Positive auto-regulation involving an autocrine loop	[15]	IL-12 auto-regulates itself through Jak/Stat pathway with STAT4 being the major transcription factor.
A	TNFα	IL-12	inhibition	[16,17]	TNFα inhibits IL-12p40 through TNFα signaling pathway.
B	IRAK4	IKK	Activation	[2]	
B	IKK	NFκB	Activation	[2]	
B	NFκB	ATF3	Transcription	[18]	
B	ATF3	C/EBPδ	Inhibition	[19,20]	
B	NFκB	IL-6	Transcription	[20]	
B	IRAK1	IKKε	Activation	[21]	
B	IKKε	C/EBPδ	Activation	[21]	Low dose LPS induces the expression of C/EBPδ

					through IRAK1 and IKKε.
B	C/EBPδ	C/EBPδ	Transcription	[20]	C/EBPδ can bind onto its own promoter to enhance the transcription.
B	C/EBPδ	IL-6	Transcription	[20]	
C	IFNγ	STAT1	Transcription	[22]	Low dose IFNγ elevates STAT1 transcription, but not STAT1 phosphorylation.
C	IFNγ	P-STAT1	Activation	[22]	Phosphorylation of STAT1 is activated only under high dose IFNγ.
C	P-STAT1	SOCS1	Transcription	[22]	
C	SOCS1	P-STAT1	Inhibit	[22]	SOCS1 inhibits the phosphorylation and activation of STAT1.
C	P-STAT1	IRF-1,<br>IP-10	Transcription	[22]	
C	P-STAT1	TNFα	Transcription	[23]	P-STAT1 may synergistically cooperate with NFκB to activate the transcription of TNFα.
C	TNFα	SOCS1	Transcription	[24]	TNFα might be able to negatively feedback on P-STAT1 through enhancing the production of SOCS1.

Table S3 References

1. O'Neill LAJ, Dunne A, Edjeback M, Gray P, Jefferies C, et al. (2003) Mal and MyD88: adapter proteins involved in signal transduction by Toll-like receptors. J of Endotoxin Res 9: 55-59.

2. Hacker H, Karin M (2006) Regulation and function of IKK and IKK-related kinases. Sci STKE 2006: re13.

3. Akira S, Takeda K (2004) Toll-like receptor signalling. Nat Rev Immunol 4: 499-511.

4. Koziczak-Holbro M, Joyce C, Glück A, Kinzel B, Müller M, et al. (2007) IRAK-4 Kinase Activity Is Required for Interleukin-1 (IL-1) Receptor- and Toll-like Receptor 7-mediated Signaling and Gene Expression. J Biol Chem 282: 13552-13560.

5. De AK, Kodys KM, Yeh BS, Miller-Graziano C (2000) Exaggerated Human Monocyte IL-10 Concomitant to Minimal TNF-α Induction by Heat-Shock Protein 27 (Hsp27) Suggests Hsp27 Is Primarily an Antiinflammatory Stimulus. J Immunol 165: 3951-3958.

6. Sica A, Saccani A, Bottazzi B, Polentarutti N, Vecchi A, et al. (2000) Autocrine Production of IL-10 Mediates Defective IL-12 Production and NF-κB Activation in Tumor-Associated Macrophages. J Immunol 164: 762-767.

7. Uyemura K, Demer LL, Castle SC, Jullien D, Berliner JA, et al. (1996) Cross-regulatory roles of interleukin (IL)-12 and IL-10 in atherosclerosis. J Clin Invest 97: 2130-2138.

8. Fiorentino D, Zlotnik A, Mosmann T, Howard M, O'Garra A (1991) IL-10 inhibits cytokine production by activated macrophages. J Immunol 147: 3815-3822.

9. Shnyra A, Brewington R, Alipio A, Amura C, Morrison DC (1998) Reprogramming of lipopolysaccharide-primed macrophages is controlled by a counterbalanced production of IL-10 and IL-12. J Immunol 160: 3729-3736.

10. Thompson AJV, Locarnini SA (2007) Toll-like receptors, RIG-I-like RNA helicases and the antiviral innate immune response. Immunol Cell Biol 85: 435-445.

11. Matsumoto M, Einhaus D, Gold ES, Aderem A (2004) Simvastatin Augments Lipopolysaccharide-Induced Proinflammatory Responses in Macrophages by Differential Regulation of the c-Fos and c-Jun Transcription Factors. J Immunol 172: 7377-7384.

12. Ma W, Gee K, Lim W, Chambers K, Angel JB, et al. (2004) Dexamethasone Inhibits IL-12p40 Production in Lipopolysaccharide-Stimulated Human Monocytic Cells by Down-Regulating the Activity of c-Jun N-Terminal Kinase, the Activation Protein-1, and NF-κB Transcription Factors. J Immunol 172: 318-330.

13. Jana M, Dasgupta S, Saha RN, Liu X, Pahan K (2003) Induction of tumor necrosis factor-α (TNF-α) by interleukin-12 p40 monomer and homodimer in microglia and macrophages. J Neurochem 86: 519-528.

14. Spriggs D, Imamura K, Rodriguez C, Horiguchi J, Kufe DW (1987) Induction of tumor necrosis factor expression and resistance in a human breast tumor cell line. P NATL ACAD SCI USA 84: 6563-6566.

15. Grohmann U, Belladonna ML, Vacca C, Bianchi R, Fallarino F, et al. (2001) Positive Regulatory Role of IL-12 in Macrophages and Modulation by IFN-γ. J Immunol 167: 221-227.

16. Zakharova M, Ziegler HK (2005) Paradoxical Anti-Inflammatory Actions of TNF-α: Inhibition of IL-12 and IL-23 via TNF Receptor 1 in Macrophages and Dendritic Cells. J Immunol 175: 5024-5033.

17. Hodge-Dufour J, Marino MW, Horton MR, Jungbluth A, Burdick MD, et al. (1998) Inhibition of interferon γ induced interleukin 12 production: A potential mechanism for the anti-inflammatory activities of tumor necrosis factor. P NATL ACAD SCI USA 95: 13806-13811.

18. Kawai T, Akira S (2010) The role of pattern-recognition receptors in innate immunity: update on Toll-like receptors. Nat Immunol 11: 373-384.

19. Gilchrist M, Thorsson V, Li B, Rust AG, Korb M, et al. (2006) Systems biology approaches identify ATF3 as a negative regulator of Toll-like receptor 4. Nature 441: 173-178.

20. Litvak V, Ramsey SA, Rust AG, Zak DE, Kennedy KA, et al. (2009) Function of C/EBP delta in a regulatory circuit that discriminates between transient and persistent TLR4-induced signals. Nat Immunol 10: 437-443.

21. Maitra U, Gan L, Chang S, Li L (2011) Low-Dose Endotoxin Induces Inflammation by Selectively Removing Nuclear Receptors and Activating CCAAT/Enhancer-Binding Protein δ. The Journal of Immunology 186: 4467-4473.

22. Hu X, Herrero C, Li W-P, Antoniv TT, Falck-Pedersen E, et al. (2002) Sensitization of IFN-[gamma] Jak-STAT signaling during macrophage activation. Nat Immunol 3: 859-866.

23. Kalliolias GD, Ivashkiv LB (2008) IL-27 Activates Human Monocytes via STAT1 and Suppresses IL-10 Production but the Inflammatory Functions of IL-27 Are Abrogated by TLRs and p38. J Immunol 180: 6325-6333.

24. Federici M, Giustizieri ML, Scarponi C, Girolomoni G, Albanesi C (2002) Impaired IFN-γ-Dependent Inflammatory Responses in Human Keratinocytes Overexpressing the Suppressor of Cytokine Signaling 1. J Immunol 169: 434-442.
