# Supplementary material for: Network Topologies and Dynamics Leading to Endotoxin Tolerance and Priming in Innate Immune Cells
Source: PLoS Comput Biol. 2012 May 17;8(5):e1002526. doi: 10.1371/journal.pcbi.1002526 (PMC3355072; doi:10.1371/journal.pcbi.1002526)
Supplement: Text S1 — Detailed explanation of parameter search algorithm, modeling methods and statistical analysis of motifs. (PDF) [file pcbi.1002526.s012.pdf]

Supporting Information

Network Topologies and Dynamics Leading to Endotoxin Tolerance and Priming in Innate Immune Cells

Table of Contents

Supporting text (Text S1):

1. Detailed criteria for priming and tolerance in the Metropolis searching algorithm.
2. Two-stage Metropolis search for parameter sets that exhibit priming or tolerance.
3. Statistical method used to identify backbone motifs.
4. Motif density is more robust than frequency to variation in the topological cut-off.
5. 2D parameter correlations demonstrate how parameter compensation affects topological robustness.

Supporting figures:

1. **Figure S1.** Illustration of the two-stage Metropolis search procedure.
2. **Figure S2.** Distribution of changes in the initial condition of x_2 between primed and non-primed system.
3. **Figure S3.** Statistical method used to identify backbone motifs from priming and tolerance data.
4. **Figure S4.** Parameter correlations highlight the backbone motifs of each priming mechanism.
5. **Figure S5.** Typical time course and corresponding trajectory in the phase space.
6. **Figure S6.** Change in the robustness rank as a result of variations in the topology cut-off.
7. **Figure S7.** Topologies of the PS and AI mechanisms.
8. **Figure S8.** Parameter correlation and compensation affects the robustness of the model.

Supporting tables:

1. **Table S1.** Criteria identifying priming and tolerance for a given parameter set x.
2. **Table S2.** Parameter sets used to generate time course and phase-space trajectory in Figure 3 and Figure S5.
3. **Table S3.** Experimental literature supporting the network details in Figure 8.

Detailed criteria for priming and tolerance in the Metropolis searching algorithm

We used the Metropolis algorithm [1] to search for parameter values for which the system exhibits priming or tolerance effects. Table S1 gives the criteria for identifying priming or tolerance parameter sets. In general, both priming and tolerance require the system to generate a dose-response curve having the following qualitative features: small signal (LD) gives small response and large signal (HD) gives large response; priming requires that LD+HD LPS gives a larger response than does a single HD LPS (positive control); tolerance requires that HD+HD LPS gives lower response than does a single HD LPS (positive control). Parameter sets that satisfy these conditions (either for priming or for tolerance) are called "good" sets.

Two-stage Metropolis search for parameter sets that exhibit priming or tolerance

It is impractical to perform a brute force search for priming/tolerance samples in a high dimensional parameter space. Figure S1A illustrates an alternative two-stage strategy. In the first stage, we searched widely over the parameter space with some bias to stay in a good parameter region and some chance to wander off in search of another good region. Then K-means Clustering and Principal Component Analysis was applied to the samples of good parameter sets generated in stage 1 to see if the data form several separate clusters. Each potential cluster provides a random seed for a second round of Metropolis searching. This time the search is restricted to stay within a good region, in order to search each region thoroughly and to obtain a representative sample of good parameter sets.

To apply the Metropolis Algorithm, we relate the current problem of searching in the parameter space to sampling the partition function of a pseudo-statistical physics system. The bias controlling the probability of wandering out of a good region ($\Omega_k = 0,\ \Omega_{k+1} = 1$) is defined by a Boltzmann-type expression $\rho = e^{-\beta(\Omega_{k+1} - \Omega_k)}$ where β represents an "inverse temperature" variable. There exists a trade-off value of β for the Metropolis search in stage I. If β is too large, the search will stay in a local minimum and fail to explore the parameter space thoroughly. If β is too small, the search cannot yield enough samples for the clustering analysis. Through trial and error, we found that β = 6 is a good value for the stage I Metropolis search, which gives ρ = 0.0025. Note that the priming region is very small compared to the whole parameter space. Therefore, although ρ = 0.0025 is very small, it still guarantees that the system has sufficient probability to leave the good regions and thoroughly search the parameter space.

In the above procedure, the score function Ω_k plays the role of "energy" in a physical system. In general it can be a continuous function, and its gradient can guide the Metropolis search to the favorable region. For the current problem, the score function we use essentially behaves as a two-state system. Therefore we assign the value of Ω_k to be 0 or 1.

We chose to use the Metropolis method for the first stage, but other methods will probably work equally well, e.g. genetic algorithm [2] and the methods used by Ma et al. [3] and Yao et al. [4].

Figure S1B provides the result of the two-stage Metropolis search. In the left panel the priming sets obtained from the first stage form three main clusters under the K-means Clustering. For visualization purpose the clusters in the high-dimensional parameter space are plotted using the first two components of Principal Component Analysis. Using the Khachiyan Algorithm [5], we calculated the minimal volume ellipsoid to embrace 99% of the parameter sets of each region. As shown in the right panel of Figure 1B which calculates the distance of a parameter set to the center of each bounding ellipsoid, it turns out that a single ellipsoid embraces clusters 1 and 2, thus forming one single region (we call it "Region I"). This result is independently confirmed with the following Metropolis simulation with $\rho = 0$: a trajectory starting from one cluster can generate parameter sets belonging to the other cluster. On the other hand, cluster 3 forms a separate region (Region II). Notice that a small portion of samples locate within both ellipsoids, indicating these two ellipsoids (regions) are barely connected. We found that Region II is actually (part of) the mirror image of Region I with the roles of x_1 and x_2 exchanged, reflecting the symmetry of the 3-node system. Therefore, the results discussed below and in the main text focus on the motifs found in Region I.

About 10^6 output samples are generated out of 10^8 Metropolis steps in stage 2. Of these 10^6 samples, some appear to be biologically irrelevant and are removed from the sample set. For example, in some cases $x_3(t)$ increases to a much higher level after the HD stimulation is removed, this would be a pathological response of the system. Other samples show unrealistically large sensitivity to initial conditions, i.e., although LD induced only small changes in x_1, x_2 and x_3 (less than 10%), the system still exhibited priming effect. If priming were due to such small differences, then (in our opinion) the response would not be robust to the stochastic fluctuation expected in real systems [6-9].

While the results reported in the main text are from one trajectory result, the procedure was repeated several times with random initial start of the searching in stage 1. Results analyzed from different trajectories agree with each other, confirming the convergence of our two-stage Metropolis searching procedure.

Statistical method used to identify backbone motifs

A backbone motif is defined to be the simplest motif (the fewest number of non-zero ω_{ji}'s) that is shared by most of the priming/tolerance network structures in a particular region. A backbone motif must be able to generate a priming/tolerance effect by itself. Identification of backbone motifs helps to define the core mechanism of priming or tolerance. Figure S3 shows the statistical method used to obtain the backbone motifs for the pathway synergy group.

Step 1: calculate the mean of each interaction coefficient ω_{ji} among all samples of the group, and map the mean values into a topological matrix τ_{ji} (see Material and methods in the main text for the method of parameter discretization).

Step 2: for each ω_{ji} calculate its coefficient of variation (CV = standard deviation divided by |mean|). The value of CV measures the dispersion of the data along each parameter dimension. A large value of CV suggests that a link is not essential and should not be part of the backbone motif. Only links with CV < *CutOff* should be part of a backbone motif. For CV > *CutOff*, $\tau_{ji} = 0$ in the backbone motif.

Step 2.1: determine the optimal value of *CutOff*. As *CutOff* decreases, the corresponding motif becomes simpler and therefore more samples contain this motif. However, the motif is a backbone motif only if it gives priming by itself. Therefore, there exists an optimal *CutOff* value so that the corresponding motif has the simplest topology that is still able to generate priming for some specific parameter sets. In this case the optimal *CutOff* = 0.54 (see the right figure in Step 2.1 of Supplement Figure 3).

Step 2.2: compare each dimension in the CV matrix to this optimal *CutOff* value, and obtain the corresponding backbone motif.

Figure S4 shows 2D histograms of parameter distributions under each priming mechanism (PS, SD and AI). These histograms clearly highlight the corresponding backbone motifs. For example, for the 2D histogram shown in Supplemental Figure 4A, the PS data form clusters where both x_1 and x_2 activate x_3 (2nd figure), and x_3 feeds back negatively on x_2 (4th figure). Also x_2 shows significant auto-activation but x_1 does not (data spread out horizontally in the 5th figure); this is in line with the backbone motif where x_1 auto-regulation is not essential for priming. Similarly, x_1 exerts strong inhibition on x_2, whereas the regulation from x_2 to x_1 can be either negative, zero or positive (the 3rd figure), in line with the backbone motif where this regulation is missing. In addition, the 1st figure indicates that x_1 should change on a much faster time-scale than x_2. This is a dynamical requirement of pathway synergy in addition to the topological features as illustrated by the backbone motif.

Motif density is more robust than frequency to variation in the topological cut-off

To map from the continuous space of interaction coefficients ω_{ji} to the discrete space of network topologies τ_{ji}, one must choose a cut-off value τ_0 for mapping ω_{ji}'s to −1, 0 or +1. We have chosen this cut-off τ_0 (somewhat arbitrarily) to be 0.1. The simplest way to order these topologies from "more robust" to "less robust" is in terms of the number of parameter sets that map into each topology, i.e., the frequency of each topology in the total data set. However, we find that topology-frequency is sensitive to the choice of the cut-off value for ω_{ji}. A better measure is topology density (Figure S6), defined as follows. The total volume of the 9-dimensional space of interaction coefficients is 2^9, because each ω_{ji} can continuously vary over [−1, 1]. For a motif with m non-zero τ_{ji}'s, the volume of its subspace is $(1-\tau_0)^m = (0.9)^m$. The density of the motif is defined as the number of samples corresponding to this motif divided by the volume of its subspace.

In Supplemental Figure 6 we compared the two ways of ordering the topologies using the SD data set as an example. The figure shows how the rank of robustness of each topology changes due to 10%, 30% and 50% positive or negative variations from the original cut-off $\tau_0 = 0.1$. A point on the figure with coordinate (x, y) means that the rank of a given topology is x with $\tau_0 = 0.1$, but y with the varied τ_0. Scattering from the diagonal indicates changing of the ranking due to τ_0 variation. The density-sorted rank (top panel) is less sensitive than the frequency-sorted one (lower panel) to the change of τ_0.

2D parameter correlations demonstrate how parameter compensation affects topological robustness

We calculated the correlation matrix of each priming mechanism from the corresponding samples. As can be seen from Figure S8A, some parameters show strong anti-correlations. For a pair of anti-correlated parameters, increasing one can be compensated by decreasing the other (or negatively increasing the other if the regulation is inhibition), so the overall dynamics remains (approximately) the same. This is because in the modeling equations,

$$\frac{dx_j(t)}{dt} = \gamma_j\left(\frac{1}{1+e^{-\sigma_j W_j}} - x_j(t)\right)$$
$$W_j = \sum_{i=1}^{3} \omega_{ji} x_i(t) + \omega_{j0} + S_j$$

the activation of species x_j is dependent on the overall net input W_j. As W_j sums inputs from all regulating nodes, a change in one parameter (e.g. ω_{j1}) can be compensated by a change in a second parameter (e.g. ω_{j2}) if the sum stays the same. Such parameter compensation expands the region of parameter space where priming or tolerance is observed and therefore affects the robustness of the model.

For example, the left panel of Supplemental Figure 8B shows that the feedback from x_3 to x_2 strongly anti-correlated with x_2's auto-regulation among SD datasets. With $\omega_{23} = 0$, the absolute value of ω_{22} needs to be also small (the Null region in the right panel of Figure 8B), otherwise priming is abolished. However, since ω_{23} and ω_{22} are anti-correlated, the effect of an increasing ω_{22} can be canceled off by increasing ω_{23}, thus expand the priming region in the parameter space (the upper left and bottom right regions of the right panel of Figure S8B).

Supporting References

1. Metropolis N, Rosenbluth AW, Rosenbluth MN, Teller AH (1953) Equation of State Calculations by Fast Computing Machines. The Journal of chemical physics 21: 1087.

2. Singhania R (2011) Modeling Protein Regulatory Networks that Control Mammalian Cell Cycle Progression and that Exhibit Near-Perfect Adaptive Responses. Blacksburg, Va: University Libraries: Virginia Polytechnic Institute and State University.

3. Ma W, Trusina A, El-Samad H, Lim WA, Tang C (2009) Defining Network Topologies that Can Achieve Biochemical Adaptation. Cell 138: 760-773.

4. Yao G, Tan CM, West M, Nevins JR, You LC (2011) Origin of bistability underlying mammalian cell cycle entry. Mol Sys Biol 7: 485.

5. Khachiyan LG (1996) Rounding of Polytopes in the Real Number Model of Computation. Math Oper Res 21: 307-320.

6. Spencer SL, Gaudet S, Albeck JG, Burke JM, Sorger PK (2009) Non-genetic origins of cell-to-cell variability in TRAIL-induced apoptosis. Nature 459: 428-432.

7. Sigal A, Milo R, Cohen A, Geva-Zatorsky N, Klein Y, et al. (2006) Variability and memory of protein levels in human cells. Nature 444: 643-646.

8. Cohen AA, Geva-Zatorsky N, Eden E, Frenkel-Morgenstern M, Issaeva I, et al. (2008) Dynamic proteomics of individual cancer cells in response to a drug. Science 322: 1511-1516.

9. Chang HH, Hemberg M, Barahona M, Ingber DE, Huang S (2008) Transcriptome-wide noise controls lineage choice in mammalian progenitor cells. Nature 453: 544-547.
